# NR4A Expression by Human Marginal Zone B-Cells

**DOI:** 10.3390/antib8040050

**Published:** 2019-10-11

**Authors:** Kim Doyon-Laliberté, Josiane Chagnon-Choquet, Michelle Byrns, Matheus Aranguren, Meriam Memmi, Pavel Chrobak, John Stagg, Johanne Poudrier, Michel Roger

**Affiliations:** 1 Centre de Recherche du Centre Hospitalier de l’Université de Montréal (CRCHUM), Tour Viger 900 rue St-Denis, Montréal, QC H2X 0A9, Canada; k.doyonlaliberte@videotron.ca (K.D.-L.); michelle.byrns@umontreal.ca (M.B.); mat.aranguren@gmail.com (M.A.); meriam.memmi@yahoo.fr (M.M.); Pavel.Chrobak@hotmail.com (P.C.); John.Stagg@umontreal.ca (J.S.); 2 Département de Microbiologie, Infectiologie et Immunologie de l‘Université de Montréal, Montréal, QC H3C 3J7, Canada; 3 Faculte de Pharmacie, Université de Montréal, Montréal, QC H3C 3J7, Canada; 4 Institut du Cancer de Montréal CRCHUM, Montreal, QC H2X 0A9, Canada

**Keywords:** NR4A, human marginal zone B-cells, B regulatory cells

## Abstract

We have previously characterized a human blood CD19^+^CD1c^+^IgM^+^CD27^+^CD21^lo^CD10^+^ innate-like B-cell population, which presents features shared by both transitional immature and marginal zone (MZ) B-cells, named herein “precursor-like” MZ B-cells. B-cells with similar attributes have been associated with regulatory potential (Breg). In order to clarify this issue and better characterize this population, we have proceeded to RNA-Seq transcriptome profiling of mature MZ and precursor-like MZ B-cells taken from the blood of healthy donors. We report that ex vivo mature MZ and precursor-like MZ B-cells express transcripts for the immunoregulatory marker CD83 and nuclear receptors NR4A1, 2, and 3, known to be associated with T-cell regulatory (Treg) maintenance and function. Breg associated markers such as CD39 and CD73 were also expressed by both populations. We also show that human blood and tonsillar precursor-like MZ B-cells were the main B-cell population to express elevated levels of CD83 and NR4A1-3 proteins ex vivo and without stimulation. Sorted tonsillar precursor-like MZ B-cells exerted regulatory activity on autologous activated CD4^+^ T-cells, and this was affected by a CD83 blocking reagent. We believe these observations shed light on the Breg potential of MZ populations, and identify NR4A1-3 as potential Breg markers, which as for Tregs, may be involved in stabilization of a regulatory status. Since expression and activity of these molecules can be modulated therapeutically, our findings may be useful in strategies aiming at modulation of Breg responses.

## 1. Introduction

We have previously reported a relatively small population (1%–2%) of B-cells expressing the surface markers CD19^+^CD1c^+^IgM^+^CD27^+^CD21^lo^CD10^+^ that is present in the blood of healthy human individuals [1,2,3]. These B-cells harbored markers of transitional immature and of innate marginal zone (MZ) B-cells, and were named “precursor-like” MZ B-cells. We previously showed that CD19^+^CD1c^+^IgM^+^CD27^+^CD21^hi^CD10^−^ mature MZ and precursor-like MZ B-cells expressed IL-10 ex vivo; precursor-like more so than mature MZ B-cells [2]. Furthermore, frequencies of IL-10 expressing precursor-like MZ B-cells were increased in the context of excess B-cell activating factor (BAFF) [2]. B-cells sharing some attributes with these precursor-like MZ B-cells have been recognized for B regulatory “Breg” activity [4]. Although the hallmark of Breg activity is IL-10 production, other regulatory factors have been depicted such as expression of PD-L1, production of TGF-β, IL-35, and adenosine via the ectonucleotidases CD39 and CD73 [4]. However, until now no precise marker can allow the identification of Bregs, as it is the case with the marker Forkhead box 3 (FoxP3) for Tregs [5].

NR4A nuclear receptors are important regulators of the inflammatory response, and can be transiently up-regulated following stimulation via the TCR or BCR [6]. However, expressions of NR4As are highly enriched in Tregs when compared to other T-cell subsets [5]. NR4As have been shown to directly promote the expression of the FoxP3 transcription factor, associated with the generation, differentiation and maintenance of Tregs [7,8]. Knockout mice in which all NR4A1-3 genes were specifically deleted in Tregs showed global reduction of FoxP3, and developed systemic autoimmune diseases, suggesting that NR4As are required for Treg cell maintenance and function [5].

We now report gene expression of nuclear receptors NR4A1, 2, and 3 as well as the immunoregulatory surface molecule CD83 [9,10], which NR4As can directly regulate [11], by both mature MZ and precursor-like MZ B-cell populations from ex vivo human blood. Moreover, we found that in human blood and tonsils precursor-like MZ B-cells are the main population to co-express NR4A1-3 and CD83 proteins at high levels without stimulation. Furthermore, we report that sorted tonsillar precursor-like MZ B-cells exert regulatory activity on autologous activated CD4^+^ T-cells, and this is affected by a CD83 blocking reagent. We also report that the Breg associated CD39 and CD73 enzymes [12,13] are co-expressed by both populations in human blood and tonsils. Total CD39^+^ B-cells from blood and tonsils also expressed NR4A1 and CD83, albeit to a lesser extent than precursor-like MZ B-cells. We believe these observations shed light on the Breg potential of MZ populations, and identify NR4A1-3 as potential Breg markers, which as for Tregs, may be involved in stabilization of a regulatory status.

## 2. Materials and Methods

### 2.1. Ethics Statement

Written informed consent was obtained from all subjects who participated in the study. The study was conducted in accordance with the Declaration of Helsinki. The methods reported in this paper were performed in accordance with the relevant guidelines and regulations and all experimental protocols were approved by the Centre Hospitalier de l’Université de Montréal (CHUM) Research Ethics Committees.

### 2.2. Cell Sorting of Human Blood MZ and Precursor-Like MZ B-Cells, RNA Isolation, and Sequencing

Peripheral blood mononuclear cells (PBMCs) from healthy donors were isolated on Ficoll gradient, re-suspended in heat-inactivated fetal bovine serum (hi-FBS) (Wisent Inc., Montreal, QC, Canada) containing 10% dimethyl sulfoxyde and stored in liquid nitrogen until use. Cells were thawed, washed in IMDM (Iscove’s Modified Dulbecco’s Medium, Gibco Life Technologies, New York, NY, USA), and processed for cell sorting with a FACSAriaIII apparatus. Live/dead exclusion was performed using LIVE/DEAD Fixable Aqua Dead cell Stain (Invitrogen Thermo Fisher Scientific, Eugene, OR, USA). Non-specific binding sites were blocked using fluorescence-activated cell sorting (FACS) buffer (1× PBS, 2% hi-FBS) supplemented with 20% hi-FBS and 10 μg mouse IgG (Sigma-Aldrich, St-Louis, MO, USA). Cells were stained using the following conjugated mouse anti-human monoclonal antibodies: PacificBlue-anti-CD19, APC-Cy7-anti-CD10 (BioLegend, San Diego, CA, USA), AlexaFluor700-anti-CD27, FITC-anti-IgM, PE-anti-CD21 (BD-Biosciences), PerCP-eFluor710-anti-CD1c (eBioscience, San Diego, CA, USA). Live CD19^+^CD1c^+^IgM^+^CD27^+^CD21^hi^CD10^−^ mature and CD19^+^CD1c^+^IgM^+^CD27^+^CD21^lo^CD10^+^ precursor-like sorted MZ B-cells were >95% pure. Total RNA was extracted using RNeasy Micro Kit (Qiagen) according to the manufacturer’s instructions. RNA integrity was validated using a RNA Pico Chip on the Agilent BioAnalyzer 2100, and RNA was sent to IRIC’s Genomics Core Facility for RNAseq transcriptomic profiling and analysis. Librairies were prepared using Clontech Ultra Low RNA SMARTer v4 (Takara) and sequenced on a HiSeq2000. Genes with false discovery rate (FDR) values <0.05 were considered to be differentially expressed. Gene expression levels were compared using raw read counts and the negative binomial distribution model implemented in DESEq2 [14], a differential expression analysis package developed for R, which adjusts for sample variations with the assumption that the vast majority of genes should have correlating expression levels. More specifically, the regularized log transformation (rlog) implemented in DESeq2 was used to transform raw data into log2 (readcount) for analysis and visualization.

### 2.3. Validation of the Cross-Reactivity of the PE-Conjugated Human Anti-Mouse Nurr77 (NR4A1) Monoclonal Antibody (mAb) 

The human REA clone anti-mouse Nurr77 (NR4A1) IgG1 antibody cross-reacts with human NR4A1 as specified by MACS Miltenyi Biotec. This was verified on total spleen cells from 8–10 weeks old female C57BL/6 mice (not shown), and on total human PBMCs (Figure S1). Cells were cultured for 3 h at 37 °C and 5% CO_2_, at a concentration of 10^6^ cells/ml in IMDM supplemented with 10^−4^ β-2-mercaptomethanol (Sigma-Aldrich), 10% hi-FBS and penicillin/streptomycin (Gibco Life technologies) in presence or absence of PMA/ionomycin (eBioscience Cell Stimulation Cocktail 500x, Invitrogen by Thermo Fisher Scientific). Cells were then harvested, washed with IMDM followed by 1× PBS and processed for flow-cytometry. Live/dead exclusion was performed using Aqua-LIVE/DEAD Fixable Stain (Invitrogen Life technologies, Eugene, OR, USA). Non-specific binding sites were blocked using FACS buffer (1× PBS, 2% hi-FBS, and 0.1% sodium azide) supplemented with 20% hi-FBS, 10 µg mouse IgG (Sigma-Aldrich, St-Louis, MO, USA) and/or 5 µg Human BD FCBlock (BD Biosciences). For human PBMCs, the following conjugated mouse anti-human monoclonal antibodies were used to detect extracellular markers: APC-mouse Anti-Human CD19 and PerCP-eFluor 710 Anti-Human CD1c (eBioscience, San Diego, CA, USA). Intra-nuclear labelling to detect NR4A1 was performed using the FoxP3/Transcription Factor Staining Buffer Set (Invitrogen by Thermo Fisher Scientific, CA, USA). Non-specific binding sites were blocked using 20% hi-FBS and 5 µg Human BD FCBlock (BD Biosciences). The PE-conjugated human REA clone anti-mouse NR4A1 was used and compared to the use of PE-conjugated human REA isotype control (MACS, Miltenyi Biotec). Cells were kept at 4 °C in 1.25% paraformaldehyde for 18 h prior to analysis. Data acquisition of 10^5^ events per sample was performed with LSRIIB (BD-Biosciences), and analysis was done with FlowJo7.6.3 software (TreeStar, Ashland, OR, USA) and GraphPad Prism. All stainings were compared to that of fluorescence minus one (FMO) values and isotype controls. Anti-mouse Ig(κ) Compbeads and CS&T Beads (BD-Biosciences) were used to optimize fluorescence compensation settings and calibrate the LSRIIB, respectively.

### 2.4. Flow-Cytometry Characterization of NR4A1, NR4A3, CD83, CD39, and CD73 Expression by Human Peripheral Blood Total, MZ, and Precursor-Like MZ B-Cells 

PBMCs were thawed, washed with IMDM followed by 1× PBS and processed for flow-cytometry. Live/dead exclusion was performed using Aqua-LIVE/DEAD Fixable Stain (Invitrogen Life technologies, Eugene, OR, USA). Non-specific binding sites were blocked using FACS buffer (1x PBS, 2% hi-FBS, and 0.1% sodium azide) supplemented with 20% hi-FBS, 10 µg mouse IgG (Sigma-Aldrich, St-Louis, MO, USA) and 5 µg Human BD FCBlock (BD Biosciences). The following conjugated mouse anti-human monoclonal antibodies were used to detect extracellular markers: APC-mouse Anti-Human CD19, BB515-mouse Anti-Human IgM, BV421-mouse Anti-Human CD10, BUV395-mouse Anti-Human CD73, BV786-mouse Anti-Human CD39, PE-Cy7-mouse Anti-Human CD83 (BD Biosciences) and PerCP-eFluor 710 Anti-Human CD1c (eBioscience, San Diego, CA, USA ). Intra-nuclear labelling was performed using the FoxP3/Transcription Factor Staining Buffer Set. Non-specific binding sites were blocked using 20% hi-FBS and 5 µg Human BD FCBlock. The PE-conjugated human REA clone anti-mouse NR4A1 was used and compared to the use of PE-conjugated human REA isotype control. The PE-conjugated mouse anti-human NR4A3 was from Santa Cruz Biotechnology. Cells were kept at 4 °C in 1.25% paraformaldehyde for 18 h prior to analysis. Data acquisition of 10^5^ events per sample was performed with LSRIIB, and analysis was done with FlowJo7.6.3 software and GraphPad Prism. All stainings were compared to that of FMO values and isotype controls. Anti-mouse Ig(κ) Compbeads and CS&T Beads were used to optimize fluorescence compensation settings and calibrate the LSRIIB, respectively. 

### 2.5. Flow-Cytometry Characterization of NR4A1, NR4A3, CD83, CD39, and CD73 Expression by Human Tonsillar B-Cells

Human tonsillar samples from healthy individuals who underwent surgical tonsillectomy were mechanically processed and cells were frozen in liquid nitrogen until use. Cells were thawed, washed in IMDM and B-cells were negatively enriched >95% by an immunomagnetic based technology (Dynabeads Untouched Invitrogen Life technologies). Total B-cells were subsequently cultured at a concentration of 10^6^ cells/ml in IMDM supplemented with 10^−4^ β-2-mercaptomethanol, 10% hi-FBS and penicillin/streptomycin, in absence or presence of stimulus (PMA/ionomycin) for 18 h at 37 °C and 5% CO_2_. Cells were recovered and processed for flow-cytometry as stated above. 

### 2.6. Functional Assays 

Autologous precursor-like marginal zone (MZ) B-cells, total CD1c- B-cells and CD4^+^ T-cells were sorted from human tonsils of healthy donors. T-cells were cultured alone or co-cultured with either of the B-cell populations at a ratio of 3:1 for 36 h at 37 °C and 5% CO_2_ on anti-CD3 (2 µg/mL) (ULTRA-LEAF Biolegend) coated flat bottomed 96 well plates with soluble anti-CD28 (2 µg/mL) (ULTRA-LEAF Biolegend), in presence or absence of an anti-CD83 blocking antibody (2 µg/mL) (HB15 cat No CBMAB-C1765-CQ Creative Biolabs). T-cell activation was assessed by flow-cytometry analyses of intra-nuclear Ki67 (eBioscience) expression following the protocol described above. 

### 2.7. Statistical Analyses 

Statistical significance of differences between groups was assessed with a unpaired Student’s T test for comparison between two groups or with an ANOVA with post-hoc Tukey test for multiple comparisons. Analyses were performed using GraphPad PRISM5.0/Windows. Results were considered significant when *p* < 0.05.

## 3. Results and Discussion

**Figure 1:**
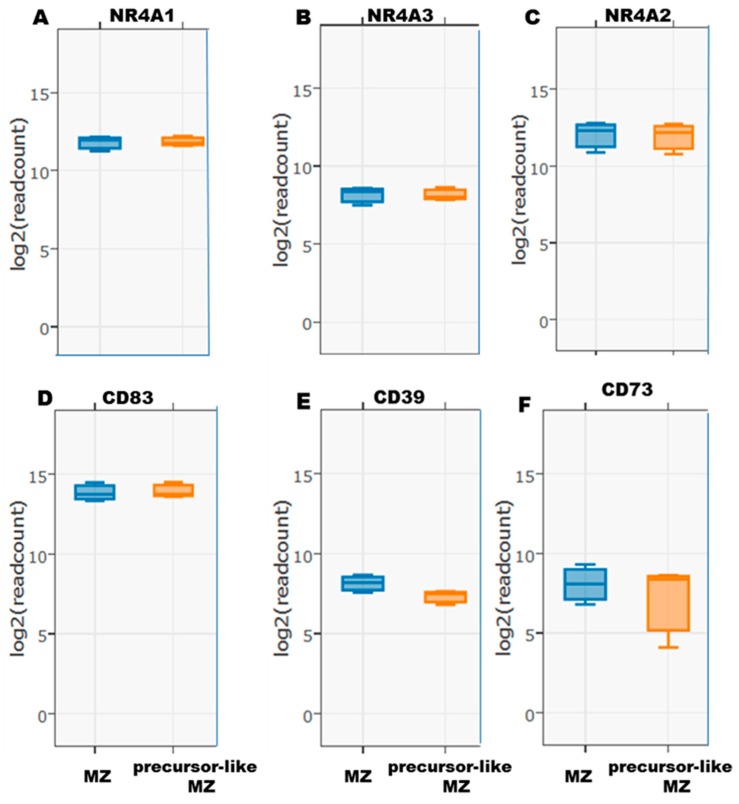
RNA-Seq analyses of (**A**) NR4A1, (**B**) NR4A3, (**C**) NR4A2, and (**D**) CD83, as well as (**E**) CD39 and (**F**) CD73 expression by ex vivo human blood marginal zone (MZ) and precursor-like MZ B-cells. Data are presented as the mean value of samples from 3 healthy donors ± SD.

Live CD19^+^CD1c^+^IgM^+^CD27^+^CD21^hi^CD10^−^ mature MZ and CD19^+^CD1c^+^IgM^+^CD27^+^CD21^lo^CD10^+^ precursor-like MZ B-cells from the blood of human healthy donors were FACS sorted. RNA-Seq transcriptomic analyses allowed us to demonstrate gene transcripts for NR4A1, 2 and 3 (Figure 1A–C), as well as for the immunoregulatory molecule CD83 in both populations (Figure 1D). Note that values obtained for the B-cell marker CD19 stand between 10 and 15 on the same log2 (readcount) scale. Gene transcripts for NR4A1 and NR4A2 were slightly more elevated than NR4A3, and those for CD83 were relatively high.

**Figure 2:**
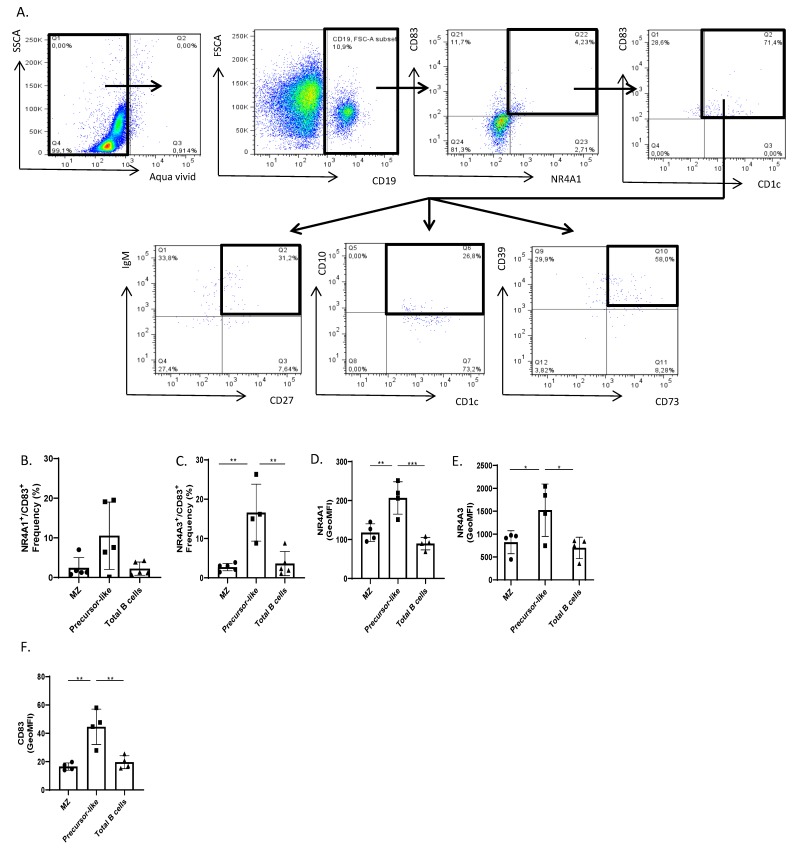
Flow-cytometry analyses of NR4A1, NR4A3, CD83, CD39, and CD73 expression by live ex vivo unstimulated human blood B-cells. (**A**) Gating strategy: Singlet Live CD19^+^ B-cells were analyzed for NR4A1 or NR4A3 and CD83 co-expression. NR4A1^+^ or NR4A3^+^ (not shown) CD83^+^ B-cells were then analyzed for CD1c expression, and subsequently for IgM and CD27 expression, for CD10 expression, and for CD39 and CD73 expression. (**B**) Relative frequencies of NR4A1 and CD83 and (**C**) NR4A3 and CD83 co-expressing marginal zone (MZ), precursor-like MZ and total B-cells were compared with an ANOVA with post-hoc Tukey test. (**A**–**C**) Data are representative of 5 healthy donors. (**D**) Levels of expression as determined by geometric mean fluorescence intensity (GeoMFI) of NR4A1, (**E**) NR4A3, and (**F**) CD83 for MZ, precursor-like MZ and total B-cells were compared with an ANOVA with post-hoc Tukey test. (**D**–**F**) Data are representative of four healthy donors. Significance levels are shown as * (*p* < 0.05), ** (*p* < 0.01), *** (*p* < 0.001).

For means of NR4A1 protein detection by flow-cytometry and because of experimental suitability, we have used the PE-conjugated human REA clonal antibody directed against murine NR4A1, which cross-reacts with human NR4A1. This was first verified on PBMCs (Figure S1). We found that stimulation with PMA/ionomycin for 3 h [6] allowed us to measure increased expression of NR4A1 by total human B-cells, of which 50% were positive for the innate glycolipid binding molecule CD1c [15] (Figure S1). Subsequently, flow-cytometry analyses of NR4A1 and CD83 protein expression on unstimulated ex vivo samples revealed that co-expression of NR4A1 and CD83 was mainly found within CD1c^+^ B-cells, which were heterogeneous and included IgM^+^CD27^+^ MZ and IgM^+^CD27^+^CD10^+^ precursor-like MZ B-cells (Figure 2A). Similar observations were found for NR4A3 and CD83 co-expression (not shown). As for the CD1c-negative B-cells which co-expressed NR4A1 and CD83, all expressed CD10 and were negative for IgM and low for CD27, reminiscent of post-germinal center B-cells, but nature of which has yet to be determined. 

Although precursor-like MZ B-cells are less frequent in blood than MZ B-cells (Figure 2A), the analyses of frequencies of NR4A1^+^CD83^+^ (Figure 2B) and NR4A3^+^CD83^+^ (Figure 2C) B-cells from five different donors show that there are more co-expressing cells within the precursor-like MZ population when compared to that of MZ and total B-cells (Figure 2B,C). Levels of expression of NR4A1, NR4A3, and CD83 were also significantly higher in precursor-like MZ B-cells when compared to MZ and total B-cells (Figure 2D–F). Albeit there is discrepancy that exists between the RNA-Seq transcript data in Figure 1 and the GeoMFI data for NR4A1, NR4A3, and CD83 in Figure 2D–F, it is important to understand that there are different major post-transcriptional mechanisms, not fully elucidated, that might interfere with a straight association between mRNA and protein levels. Furthermore, this can change from gene to gene.

The hydrolysis of extracellular ATP by membrane-bound ectonucleotidases CD39 and CD73 confer Breg potential [13,16] by allowing production of immunosuppressive adenosine. Signaling via the adenosine receptor has been shown to regulate NR4A expression [17]. As such, RNA-Seq (Figure 1E,F) analyses of blood mature MZ and precursor-like MZ B-cells revealed expression of both CD39 and CD73 by these populations. Flow-cytometry analyses of CD1c^+^ B-cells co-expressing NR4A1 and CD83, which include MZ populations, revealed the majority expressed CD39 and a substantial fraction were also CD73^+^ (Figure 2A). On the other hand, flow-cytometry analyses starting from blood MZ populations revealed that not all of these cells expressed CD39 and CD73 proteins, and not all that are CD39^+^CD73^+^ co-expressed NR4A1and CD83. However, those that did co-express NR4A1 and CD83 were in majority CD39^+^CD73^+^ (Figure S3C). 

**Figure 3:**
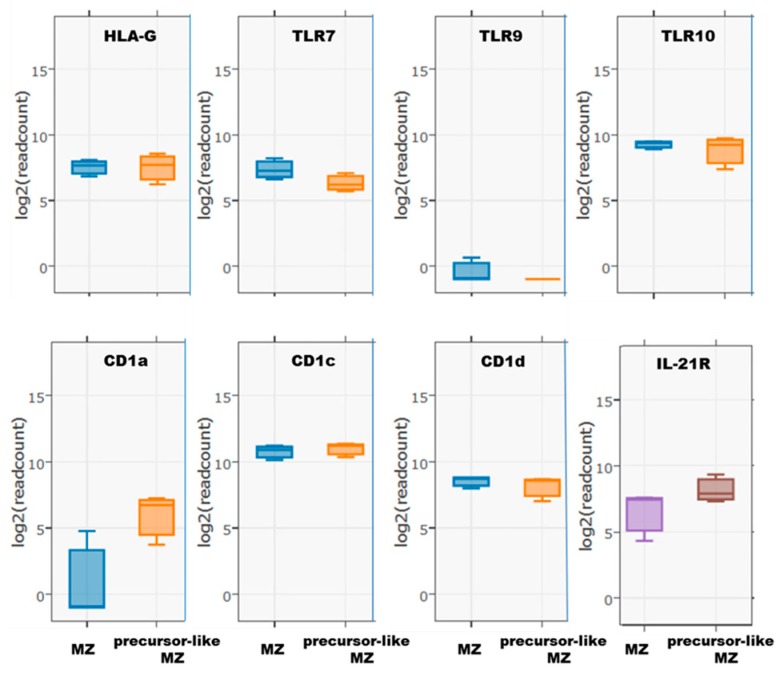
RNA-Seq analyses of HLA-G, TLR7, 9, 10, and CD1a, c, d, and IL-21R expression by ex vivo human blood marginal zone (MZ) and precursor-like MZ B-cells. Data are presented as the mean value of samples from 3 healthy donors ± SD.

RNA-Seq analyses also showed that MZ and precursor-like MZ B-cells express other Breg associated molecules [18] (Figure S2). Moreover, MZ and precursor-like MZ B-cells express molecules often associated with regulatory functions such as HLA-G [19] and TLR10 [20,21] (Figure 3). Of note, both populations did not express transcripts for TLR9, and rather expressed TLR7 (Figure 3). RNA-Seq detected CD1d transcripts in both populations (Figure 3), the latter which is expressed by most Bregs in mice and in humans [18], and which has recently been shown to be important for induction of iNKT cell suppressive functions, identifying another way by which Bregs may exert regulatory functions [22]. Interestingly, we found CD1a and IL-21R gene transcripts mainly in precursor-like MZ B-cells (Figure 3), and IL-21 has been shown to modulate B-cell suppressive functions [23]. Although appealing, a role for IL-21 in modulating Breg activity of precursor-like MZ B-cells remains to be established. 

**Figure 4:**
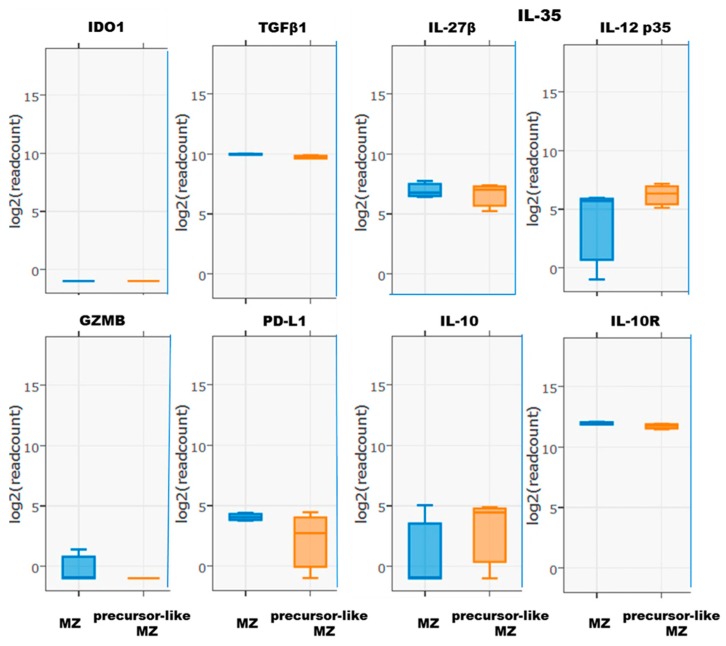
RNA-Seq analyses of Indoleamine 2, 3-dioxygenase 1 (IDO1), TGFβ1, IL-35, granzyme B, PD-L1, IL-10, and IL-10R expression by ex vivo human blood marginal zone (MZ) and precursor-like MZ B-cells. Data are presented as the mean value of samples from 3 healthy donors ± SD.

RNA-Seq demonstrated relatively high levels of gene transcripts for TGF-β1 and IL-10R in both B-cell populations (Figure 4). IL-35 and IL-10 gene transcripts were lower, which could be consistent with the capacity to produce these cytokines upon stimulation [18]. We also found several molecules associated with Breg activity, such as Indoleamine 2, 3-dioxygenase 1 (IDO1) and granzyme B (GZMB) [18] that were not expressed neither by blood mature MZ nor precursor-like MZ B-cells ex vivo (Figure 4). 

**Figure 5:**
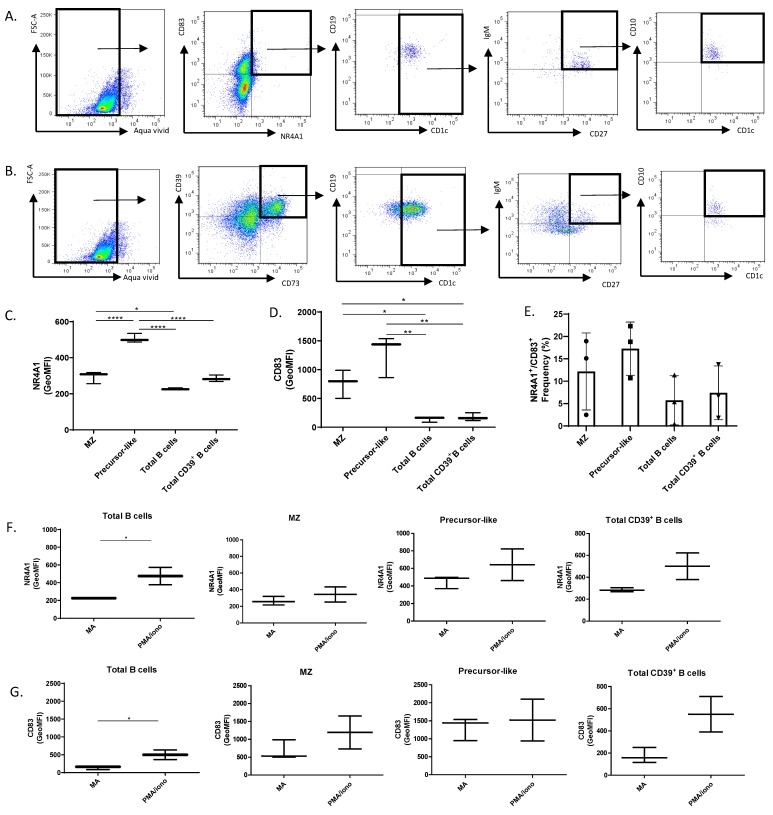
Flow-cytometry analyses of live unstimulated human tonsillar B-cells. (**A**) B-cells which co-expressed NR4A1 and CD83 were further analyzed for CD1c^+^ expression, the latter were then analyzed for IgM and CD27 expression, and IgM^+^CD27^+^ cells were analyzed for CD10 expression. (**B**) B-cells which co-expressed CD39 and CD73 were analyzed for CD1c^+^ expression, the latter were then analyzed for IgM and CD27 expression, and IgM^+^CD27^+^ cells were analyzed for CD10 expression. (**C**) Levels of expression of NR4A1, (**D**) CD83 for total, MZ, precursor-like MZ and total CD39^+^ B-cells, and (**E**) Frequencies of NR4A1 and CD83 co-expressing total, MZ, precursor-like MZ and total CD39^+^ B-cells were compared with an ANOVA with post-hoc Tukey test. (**F**) NR4A1 and (**G**) CD83 expression levels for total, MZ, precursor-like MZ and total CD39^+^ B-cells following stimulation or not with PMA/ionomycin were compared with an unpaired Student’s t test. Data are representative of at least 3 different donors (**A**,**B**). Data are presented as mean value of 3 independent experiments for the same donor ± SD (**C**–**G**), and was repeated for at least 3 healthy donors. Geometric Mean fluorescence Intensity (GeoMFI), Medium Alone (MA). Significance levels are shown as * (*p* < 0.05), ** (*p* < 0.01), **** (*p* < 0.0001).

In contrast to what was found in blood, precursor-like MZ B-cells were more abundant than MZ in human tonsils (Figure S3A). Reminiscent of our observations in blood, we found that when unstimulated, precursor-like MZ B-cells were the main population co-expressing the NR4A1 and CD83 proteins (Figure 5A,E), and levels of expression of NR4A1 and CD83 were significantly higher in precursor-like MZ B-cells when compared to total, total CD39^+^ or MZ B-cells (Figure 5C,D). Similar preliminary observations were found for NR4A3^+^CD83^+^ B-cells (Figure S3B). Unstimulated live B-cells which co-expressed CD39 and CD73 were mostly CD1c^+^ B-cells, which included IgM^+^CD27^+^CD10^−^ MZ and CD10^+^ precursor-like MZ B-cells (Figure 5B). Flow-cytometry analyses of precursor-like MZ B-cells revealed that not all these cells expressed CD39 and CD73, but it is within the latter that we found the highest proportion of NR4A1^+^CD83^+^ cells (Figure S3A). Following stimulation with PMA/ionomycin, NR4A1 and CD83 expression levels were significantly upregulated in total B-cells (Figure 5F,G left panels) and there was a strong trend for increased expression by total CD39^+^ B-cells (Figure 5F,G right panels), but levels of expression by MZ populations were less significantly affected (Figure 5F,G middle panels) presumably in relation to their already elevated expression levels ex vivo.

**Figure 6:**
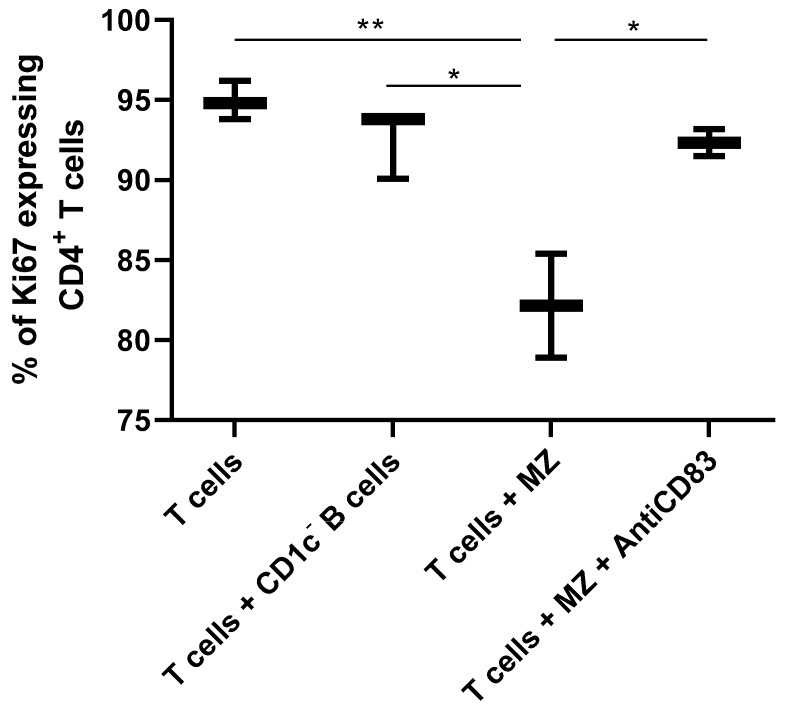
Reduced CD4^+^ T-cell proliferation following co-culture with precursor-like MZ B-cells. Sorted tonsillar precursor-like MZ or total CD1c- B-cells were co-cultured for 36 h with anti-CD3 + anti-CD28, with autologous sorted tonsillar CD4^+^ T-cells, at a ratio of 3:1 T:B cells, in presence or absence of an anti-CD83 blocking antibody. Note that because of limited tonsillar samples, we could not assess ratios above 3:1. Data are presented as percentages (%) of Ki67 (cell cycle) expressing T-cells and % between groups were compared with an ANOVA with post-hoc Tukey test. The data are presented as mean values of triplicates ± SEM for one donor and are representative of two independent donors. Anti-CD83 blocking was done once. Significance levels are shown as * (*p* < 0.05), ** (*p* < 0.01).

Finally, we have assessed the capacity of tonsillar precursor-like MZ B-cells to regulate T-cell activation in vitro. We show that percentages of activated T-cells expressing Ki-67 (Figure 6) are reduced (approximately 15%) following co-culture at a 3:1 ratio with precursor-like MZ B-cells, but not in presence of CD1c- B-cells. These findings are similar to what has been reported for Breg activity at similar T:B ratios [24]. Furthermore, this regulatory function was affected in presence of an anti-CD83 blocking antibody (Figure 6). Given the low yield of precursor-like MZ B-cells, studies with an isotype control have been performed with CD1c- B-cells co-cultured with autologous T-cells on anti-CD3 + anti-CD28. Both anti-CD83 and the isotype control had no effect on T-cell Ki-67 expression.

Altogether, our data show that human blood MZ and precursor-like MZ B-cells express NR4A1, 2 and 3 as well as high levels of CD83 gene transcripts. They also express CD39 and CD73 gene transcripts. However, we found that not all blood MZ and precursor-like MZ B-cells express proteins for these markers. Co-expression of NR4A1 or NR4A3 and CD83 proteins is heterogeneous, and the majority of B-cells co-expressing these markers are CD1c^+^ and express CD39, of which a proportion is also CD73^+^. Strikingly, elevated frequencies of cells co-expressing NR4A and CD83 are found within the precursor-like MZ B-cell population. Moreover, it is within the latter that we found significantly elevated expression levels of these proteins. As for blood, we found that in tonsils the main population to co-express NR4A and CD83 are precursor-like MZ B-cells, which bear the highest levels of expression for these markers. We found that cells co-expressing NR4A1 and CD83 are mostly CD39^+^CD73^+^ but not all CD39^+^CD73^+^ cells express NR4A1 and CD83. We found that sorted total precursor-like MZ B cells exerted regulatory activity on activated autologous tonsil CD4^+^ T-cells, and this activity was reduced by an anti-CD83 blocking antibody. 

Breg potential within precursor-like MZ B cells might be found within an NR4A and CD83 co-expressing sub-population or differentiation stage. However, further experimentation would be required to ascertain this point, and we feel it is beyond the scope of this manuscript.

MZ populations are known for their polyreactive BCR and its autoreactive potential [24,25]. It is therefore possible that NR4A1-3 expression in these cells be maintained similarly to that observed for Tregs, where NR4A1-3 expression is likely maintained through a tonic autoreactive TCR signal [5]. Despite the requirement for further experimentation, our data suggest that maintenance of NR4As expression allows for high CD83 expression, which regulatory properties could be important to Breg function. Given that activated tonsillar T-cells expressed CD83, we propose that homotypic interactions with high CD83 on precursor-like MZ B-cells might have delivered regulatory signals, as has been described for dendritic cells whereby CD83 interactions via cell to cell contact mediated inhibition of pro-inflammatory signaling by inhibiting p38α phosphorylation [26]. This however will require further experimentation.

## 4. Conclusions

We believe these observations shed light on the Breg potential of MZ populations, mainly within the precursor-like fraction and identify potential Breg markers NR4A1-3 and CD83, which as for Tregs, may be involved in stabilization of a regulatory status. Our findings may be useful to therapeutic strategies aiming at modulation of Breg responses.

## Supplementary Materials

The following are available online at https://www.mdpi.com/2073-4468/8/4/50/s1, Figure S1: NR4A1 expression by human blood B-cells following 3 h incubation in absence or presence of PMA/ionomicin. Figure S2: RNA-Seq analyses of expression of Breg associated molecules by ex vivo human blood marginal zone (MZ) and precursor-like MZ B-cells. Figure S3: Flow-cytometry analyses of blood and tonsil marginal zone (MZ) B-cells.
