# Supplementary material for: NR4A Expression by Human Marginal Zone B-Cells
Source: Antibodies (Basel). 2019 Oct 11;8(4):50. doi: 10.3390/antib8040050 (PMC6963983; doi:10.3390/antib8040050)
Supplement: Supplementary file 1 [file antibodies-08-00050-s001.pdf]

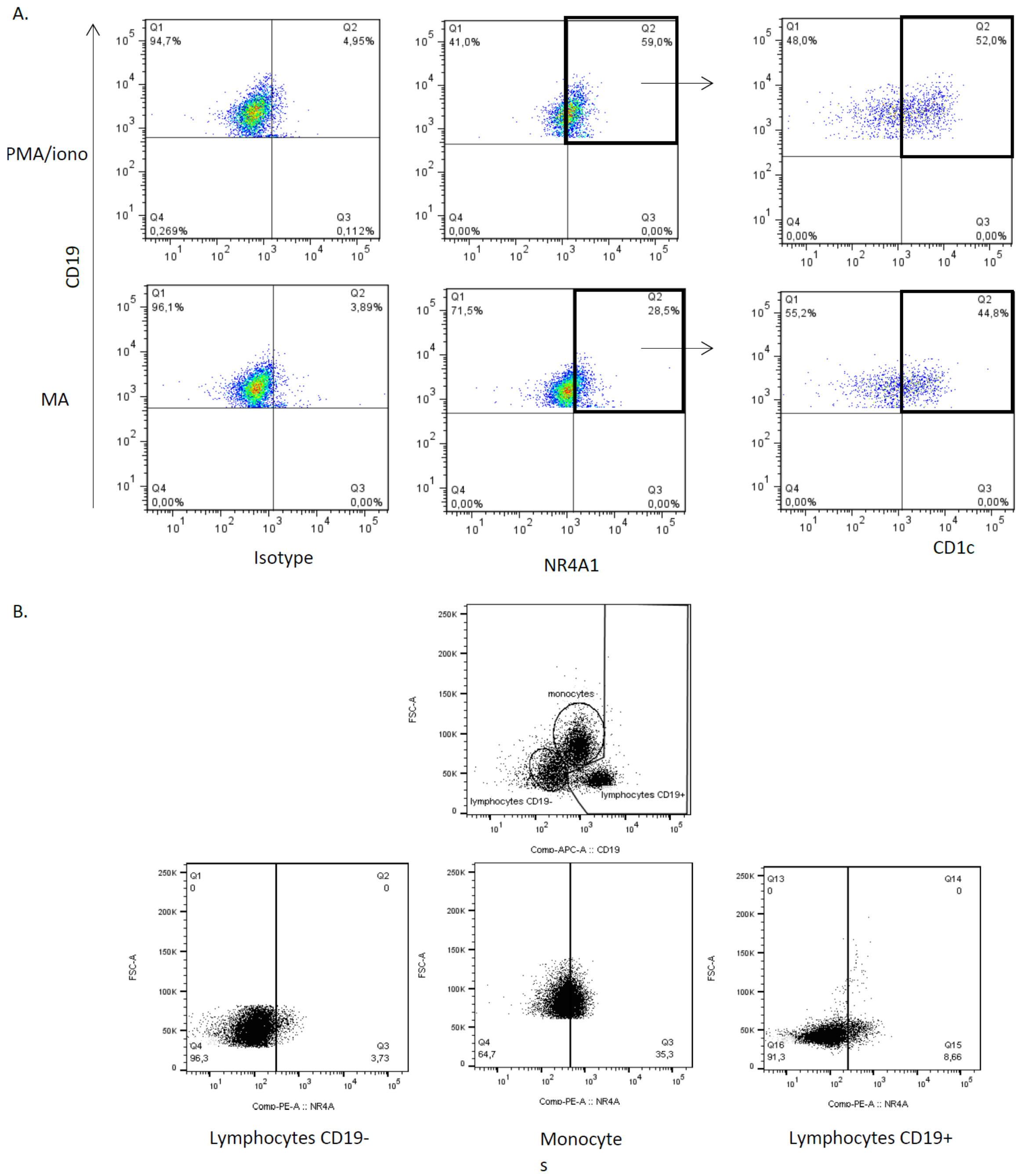

Supplementary Figure S1. (**A**) NR4A1 expression by human blood B-cells following 3 h incubation in absence or presence of PMA/ionomycin. The REA human IgG1 clone directed against mouse NR4A1 (Nurr77) cross-reacts with human, as specified by Miltenyi Biotec. (**B**) This has been verified by flow-cytometry on ex vivo human peripheral blood mononuclear cells. Data are representative of at least 3 independent experiments. Medium alone (MA).

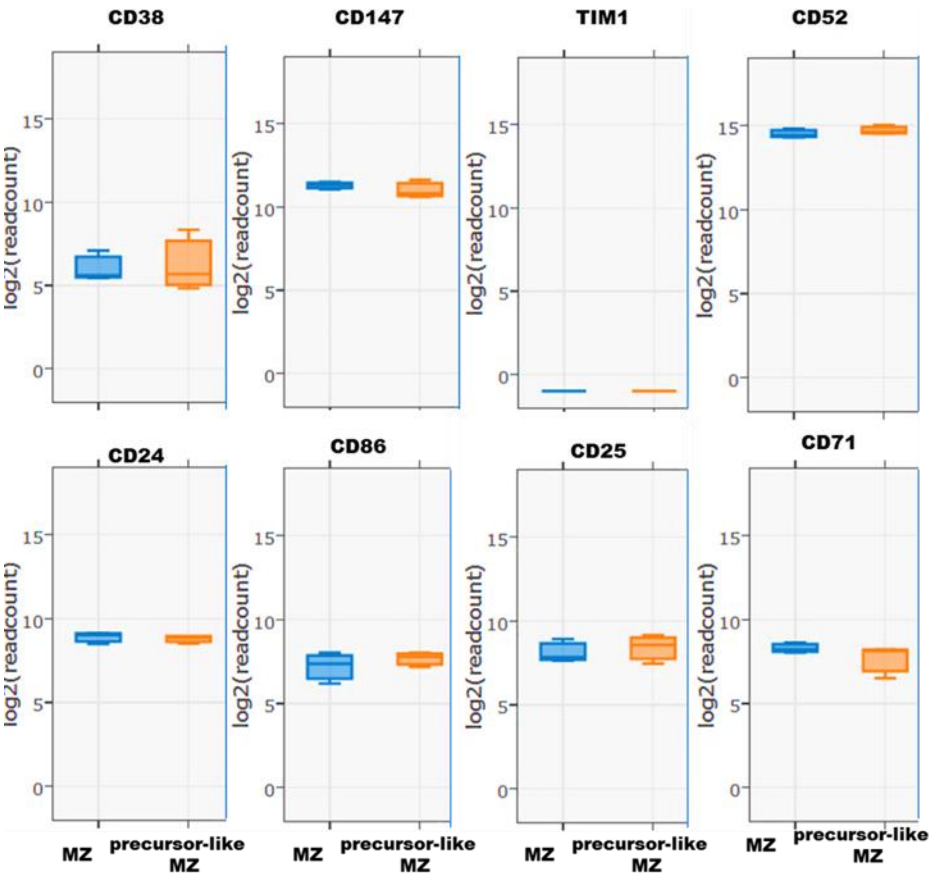

Supplementary Figure S2. RNA-Seq analyses of expression of Breg associated molecules by *ex vivo* human blood marginal zone (MZ) and precursor-like MZ B-cells. Data are presented as mean value for 3 healthy donors ± SD.

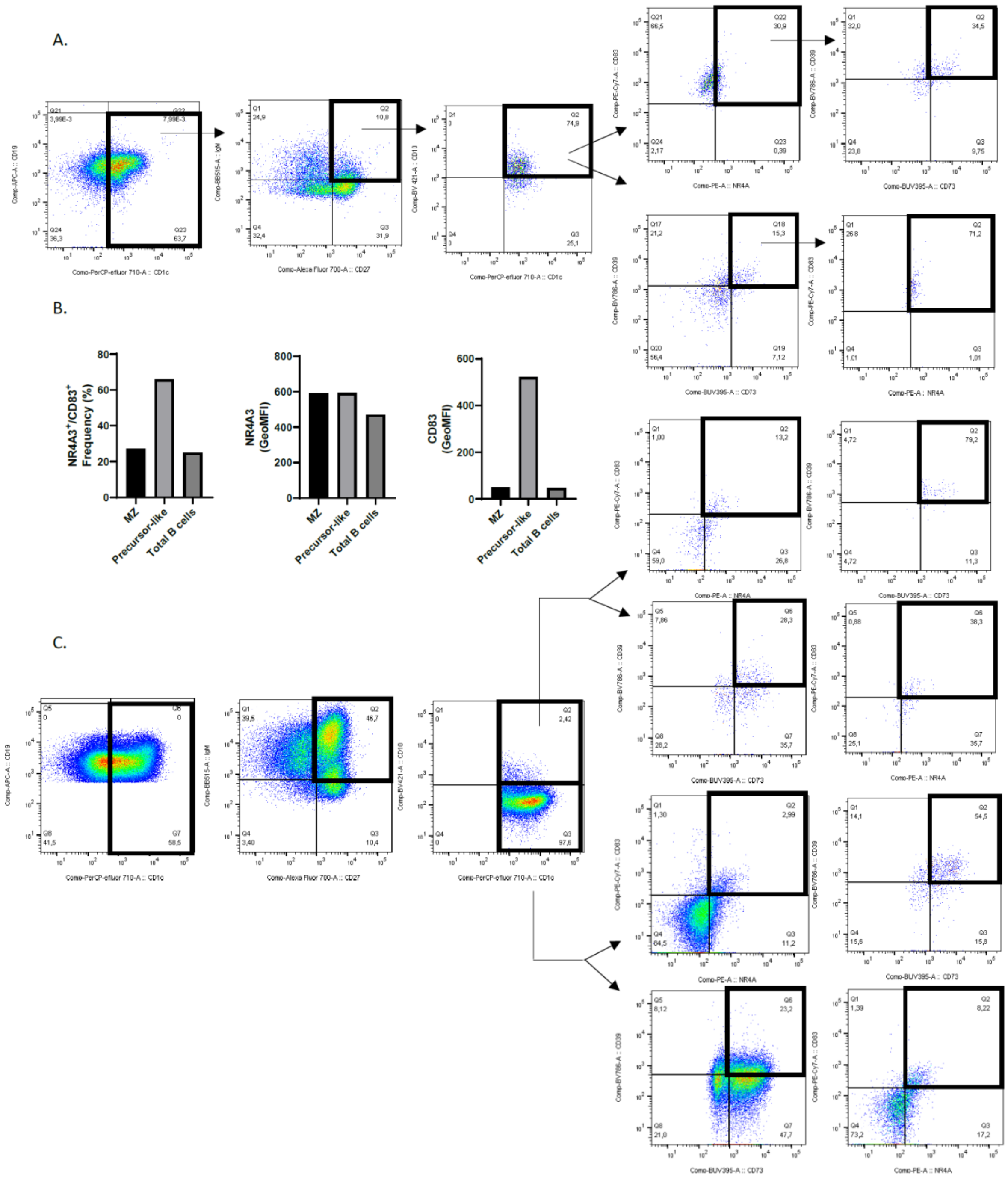

Supplementary Figure S3. Flow-cytometry analyses of blood and tonsil marginal zone (MZ) B-cells. (**A**) Tonsil ex vivo unstimulated precursor-like MZ B-cells were analyzed for expression of NR4A1 and CD83, and cells co-expressing NR4A1 and CD83 were analyzed for their expression of CD39 and CD73 (top panels). On the other hand, precursor-like MZ B-cells were analyzed for expression of CD39 and CD73, and double positive cells were assessed for NR4A1 and CD83 co-expression (lower panels). Data are representative of 3 healthy donors. (**B**) Flow-cytometry analysis of NR4A3 and CD83 co-expression by tonsil ex vivo unstimulated MZ, precursor-like and total B-cells. Data is preliminary and representative of one healthy donor. (**C**) Blood ex vivo unstimulated MZ and precursor-like MZ B-cells were analyzed for expression of NR4A1 and CD83, and cells co-expressing NR4A1 and CD83 were analyzed for their expression of CD39 and CD73 (top panels).On the other hand, MZ and precursor-like MZ B-cells were analyzed for expression of CD39 and CD73, and double positive cells were assessed for NR4A1 and CD83 co-expression (lower panels). Data are representative of 5 healthy donors.
